# Sources of Propylene Glycol and Glycol Ethers in Air at Home

**DOI:** 10.3390/ijerph7124213

**Published:** 2010-12-15

**Authors:** Hyunok Choi, Norbert Schmidbauer, John Spengler, Carl-Gustaf Bornehag

**Affiliations:** 1 Department of Environmental Health, Harvard School of Public Health, USA; E-Mails: hchoi@hsph.harvard.edu (H.C.); spengler@hsph.harvard.edu (J.S.); 2 Norwegian Institute for Air Research, P.O. Box 100, NO-2027 Kjeller, Norway; E-Mail: norbert.schmidbauer@nilu.no; 3 Public Health Sciences, Karlstad University, SE-651 88 Karlstad, Sweden; 4 SP Technical Research Institute of Sweden, Boras, Sweden

**Keywords:** indoor, volatile organic compound, glycol ether, asthma, solvent, children

## Abstract

Propylene glycol and glycol ether (PGE) in indoor air have recently been associated with asthma and allergies as well as sensitization in children. In this follow-up report, sources of the PGEs in indoor air were investigated in 390 homes of pre-school age children in Sweden. Professional building inspectors examined each home for water damages, mold odour, building’s structural characteristics, indoor temperature, absolute humidity and air exchange rate. They also collected air and dust samples. The samples were analyzed for four groups of volatile organic compounds (VOCs) and semi-VOCs (SVOCs), including summed concentrations of 16 PGEs, 8 terpene hydrocarbons, 2 Texanols, and the phthalates n-butyl benzyl phthalate (BBzP), and di(2-ethylhexyl)phthalate (DEHP). Home cleaning with water and mop ≥ once/month, repainting ≥ one room prior to or following the child’s birth, and “newest” surface material in the child’s bedroom explained largest portion of total variability in PGE concentrations. High excess indoor humidity (g/m^3^) additionally contributed to a sustained PGE levels in indoor air far beyond several months following the paint application. No behavioral or building structural factors, except for water-based cleaning, predicted an elevated terpene level in air. No significant predictor of Texanols emerged from our analysis. Overall disparate sources and low correlations among the PGEs, terpenes, Texanols, and the phthalates further confirm the lack of confounding in the analysis reporting the associations of the PGE and the diagnoses of asthma, rhinitis, and eczema, respectively.

## 1. Introduction

Inhalation exposure to evaporated components of the consumer products, building structural materials as well as their secondary reactive products at home has been associated with increased risks of asthma-like symptoms, asthma diagnosis, as well as other allergic symptoms in both adults [1] and children [2,3]. In particular, occupational and non-occupational exposure to volatilized components of water-based paint, water-based cleaning products, glass cleaning, oven cleaning, dish-washing, and the use of chlorine bleach significantly increases the risks of self-reported asthma, clinically diagnosed asthma, and other acute respiratory symptoms [1,4–13]. However, specific compounds underlying these illnesses due to such consumer product uses have remained overall unclear [13,14]. In addition, the mechanisms by which volatile organic compounds (VOCs) or semi-VOCs (SVOCs) act as sensitizers or respiratory irritants are not obvious [13].

In our recent case-control investigation of the children between the ages 3 and 8, those within the top 25 % for the level of the summed 16 PGEs in indoor air had a 130 % higher likelihood of multiple allergic symptoms (*i.e.*, parental report and clinically validated asthma, rhinitis and eczema) (95% CI, 20–370%), a 100% higher likelihood of asthma diagnosis (95% CI, −10–340%), a 320% higher likelihood of rhinitis diagnosis (95% CI, 70–930%), a 150% higher likelihood of eczema diagnosis (95% CI, 10–430%), and a 120% higher likelihood of being IgE-sensitized among the cases only (95% CI, −10–480%), accounting for gender, secondhand smoke, allergies in both parents, wet cleaning with chemical agents, residence construction period, limonene, cat and dog allergens, butyl benzyl phthalate (BBzP), and di(2-ethylhexyl)phthalate (DEHP) [15].

Water-based cleaning products either applied with an applicator (e.g., mop) or sprayed as solution can release a number of glycol ethers, terpenes, and terpene alcohols to air [16]. Major volatile organic compounds emitted from the water-based paint include propylene glycol, other glycols, glycol ethers, and Texanols (also known as TXIB™ or 2,2,4-trimethyl 1,3-pentanediol monoisobutyrate) [17]. The water-based paint also emits smaller amounts of isobutanol, toluene, xylene, trimethylbenzenes, n-nonane, n-decane, and n-undecane [17]. Due to their effectiveness as solvents, propylene glycol and glycol ethers (PGEs) are also used in pharmaceuticals, pesticides, cosmetics, varnishes, inks, pigments, adhesives, detergents, agrochemicals, and processed foods [16,18]. For other purposes, they are used in polyvinyl chloride (PVC) pipes [19], hydraulic and brake fluids, de-icing fluids for aircrafts, and artificial theatrical smoke [20].

It has been known for more than three decades that inhalation of several vaporized PGE compounds, including *α*- and *β*- isomers of propylene glycol monomethyl ether (PGME), induces airway and ocular irritation of humans [17,21,22] and rats [23]. Several experimental investigations of healthy adult volunteers have demonstrated that an administration of propylene glycol, and a mixed vapour of glycol ethers and Texanol, could respectively induce acute eye, nose, throat irritation and dyspnea [7,17,22,24]. In a prospective cohort study of house-painters, an exposure to water-based paint led to a significantly higher incidence of chest tightness/wheezing, airway irritation, bronchial hyper-responsiveness, and shortness of breath [6]. Within a non-occupational setting, greater likelihood of asthma symptoms have been observed in adults exposed to a newly painted wood or kitchen surfaces [25], and/or synthetic material-based furniture [26,27].

However, one of the considerable challenges regarding the assessment of human health risk of PGEs stems from the correlated nature of the VOC compounds emitted from multiple sources within the indoor environment. Furthermore, the role of other indoor environmental conditions (e.g., humidity, ventilation, or temperature) on the emission and/or retention of these PGEs are poorly understood [28]. Human exposure scenarios at home are likely to be different from the emission characteristics within controlled experimental setting. For example, glycol ethers emitted from completely assembled flooring materials within a completely built structure was > 100-fold higher than the levels emitted by the individual components in a laboratory chamber [29].

Here, we examined the sources and interactions of four VOC groups commonly found in water-based paints and/or cleaning products in homes, including PGEs (16 compounds), terpenes (8 compounds), Texanols (2 compounds), and phthalates (2 compounds). The PGEs, Texanols [15], and phthalates [2,30] have recently been identified as potential contributors of asthma and allergies. In addition, terpene hydrocarbons and their alcohols are investigated here because they represent common scenting agents in cleaning products [31,32]. Their reaction with ozone could induce upper airway and eye irritation [31,32]. Specific goals were to: a) examine the human activities and sources that predict the PGE levels in air; and b) investigate correlation of home indoor PGEs with other compounds emitted from cleaning and wood based materials (e.g., terpenes), home structural material and consumer products (e.g., phthalates), and paints (e.g., Texanols).

## 2. Experimental Section

### 2.1. Exposure Assessment

Three hundred ninety homes the children participating in a nested case-control investigation of asthma and allergies were inspection and air and dust samples were collected [15]. Professional inspectors visually examined the homes for the water damages, the presence of mold odour, building’s structural characteristics, indoor temperature, relative humidity, absolute humidity difference between indoor and outdoor air (termed excess indoor humidity from here on), and air exchange rate [33]. The building inspectors also examined the housing demographics (*i.e.*, type, age, quality, and ventilation system), urbaneness of the neighborhood, indoor combustion sources, and perception of air quality. Inspectors recorded wall surface materials (*i.e.*, wall paper, plastic covered wall-paper, painted glass fiber, wooden panel, tile, wooden fabric, or other) as well as the flooring material (*i.e.*, linoleum, PVC carpet, parquet, laminate, soft carpet, cork and plastic carpet, stone, or other). The type of home foundation, quality of home basement, crawl space, outer wall material, and building façade material were also examined. Excess indoor humidity was measured as a difference between indoor and outdoor water vapor content (g/m^3^). The water vapor content was higher indoors relative to the outdoors in 342 of 343 homes in which the measurement was taken. Home inspectors were unaware of the health outcome status of the child in the homes they were inspecting. Ventilation rates were measured using a passive perfluorocarbon tracer (PFT) gas method, as described in NT VVS 118 [34,35]. The PFT method measured multiple (≤ 12) sources depending on the size of the home with multiple (≤5) collectors. Separate mean ventilation rates were calculated for the entire house/week as well as the child’s bedroom/week.

Samples of dust from 390 homes were collected from moldings and shelves in the children’s bedroom with a hand-held vacuum. The dust was collected onto 90-mm membrane filters made of pure cellulose in holders made of styrene-acrylonitrile polymer mounted on a sampler made of polypropylene (VacuuMark disposable nozzle; Petersen Bach, Bjerringbro, Denmark) connected to a vacuum cleaner [36]. Of the 390 homes, 362 dust samples met the quality assurance criteria [30]. To further ensure reliability, only the dust samples with a measurable net increase in weight (≥25 mg) are included in the present study [30].

#### 2.1.1. Air Sampling

Single VOC sample was taken by placing the air sampler 1 meter above the floor in the room. A SKC pocket pump (SKC Pocket Pump 210–1002, SKC Blandford, Dorset, UK) drew in air at 80 mL/min for 60 to 90 minutes (5 to 8 liters) through a Perkin Elmer adsorption tubes (glass, 300 mg Tenax TA). The tubes were sealed with PTFE stoppers, wrapped in alumina foil and kept at −20 °C until they were shipped to NILU, Norway for analysis within two weeks of collection. Prior to use the Tenax tubes were cleaned using thermo-desorption at 275 °C for 15 minutes for three consecutive cleaning cycles. Use of adsorbent, preparation of adsorbent tubes, sampling equipment, sampling flow and safe-sampling volumes, analytical methods and analytical equipment followed international standards on ambient air quality DIN EN 14662-1 (DIN ISO 5725-2 and 3). There is no standard procedure for VOC sampling and analysis for indoor air, but the chosen method is widely accepted for its reliability [37].

Tenax TA for indoor air analysis is well established with inter-laboratory differences reported to be <10% for benzene analysis (DIN EN 14662-1 annex H2). The temporal stability of compounds trapped on Tenax TA and the formation of artifacts from degradation of the adsorbent Tenax TA is widely discussed in literature [31,38,39]—the main degradation products are known as Benzaldehyde, Acetophenone and Benzoic acid and to a minor extent Benzene, Toluene and Xylenes. Other artifacts include aldehydes (Hexanal, Heptanal, Octanal, Nonanal, Decanal), created due to the reactions of ozone from the sample air and fatty acids. All those compounds are also common gases in indoor air. The blank values of those compounds are small compared to the amount of those gases in indoor air with a sample size of more than 5 liter. Due to the chemical structure of Tenax TA (2,6-diphenylene based polymer), formation of glycol ethers as artifact from Tenax TA is very unlikely and has never been reported.

#### 2.1.2. Laboratory Analysis

The VOC samples were analyzed using an automated thermo desorption unit (Perkin Elmer ATD 400, Perkin Elmer Inc., Waltham, MA, USA) followed by GC-MS [31,38,39]. The samples were desorbed at 250 °C—refocused on a Tenax TA cold trap held at minus 30 °C and transferred to the GC-MS at 225 °C. A Hewlett Packard G 1800 A GC-MS was used as detector maintained at 250 °C. The separation column (DB-1701, 32 m length, 1 ≤m film, 0.32 mm in diameter) was programmed from 40 °C to 250 °C. The mass range of the detector was from mass 33 to mass 350. Further details on detection, calibration and quality assurance assessment are described in Online Supporting Documents.

### 2.2. Statistical Analysis

#### 2.2.1. Descriptive analysis

In every air sample, the 50 compounds with the highest concentrations were reported as Toluene-equivalents [31,38,39]. In addition, the number of all compounds within each sample with a concentration above a baseline-threshold of 0.1 ppb (usually between 180 and 250 compounds) and the concentration-sum of all those compounds were reported [31,38,39]. This resulted in an identification of 405 compounds from 381 homes (excluding ten siblings and nine missing samples). Of 405 compounds, we restricted the compound groups of interest as four classes: summed concentrations of 16 PGEs, 8 terpene hydrocarbons (*i.e.*, markers for water-based cleaning), Texanol A and B (*i.e.*, markers for water-based paint), and phthalates (BBzP and DEHP). These four groups of compounds have been identified as independent risk factors of asthma and allergies [28,30]. As PGEs constitute main compounds of interest, investigating the degree of mutual correlations according to the sources and human behaviors were necessary, in order to clarify the independent risks of PGEs on asthma and allergies.

Our earlier validation analysis demonstrated that the non-reported concentrations of the compounds are likely to be lower than the 50th compound concentration range (0.33–11.24 μg/m^3^) of the present investigation [15]. Rather than attributing the non-reported concentrations with one-half of the median (1.11 μg/m^3^) of the lowest known concentrations across all samples, we restricted our analysis to the compounded with quantified concentration. This assumes that the non-reported concentrations of the compounds are below 0.33 μg/m^3^. Validity of this assumption is supported by the analysis of similar data in Finland [40]. All compounds were natural log(ln)—transformed, considering their right skewed distributions and varying standard deviations. Following the transformation, the distribution of the four chemical groups approximated the normal distribution (all Komogorov-Smirnov tests > 0.05) with comparable standard deviations. The geometric mean concentration patterns of the four groups of compounds were compared with the other chemical groups. Considering that the multiple comparisons of the compounds would increase the likelihood of a chance association, we defined Bonferroni corrected α = 0.00625 (shown in Table 2). Test for linear trend was conducted with linear-by-linear chi-square test. In order to ensure high reliability of the phthalates in the dust sample, the dust samples with weight > 25 mg are included for all analysis pertaining to BBzP and DEHP [30].

#### 2.2.2. Predictive model of indoor PGE concentration

In order to clarify whether the PGEs, terpenes and the Texanols are emitted from similar sources or modified by common building factors, an identical predictive model was used to examine its ability to explain the respective variability in the three groups of VOCs (Table 3). We conducted multivariate, ordinary least square regression after stratifying the data according to the median excess indoor humidity (2.158 g/m^3^). In order to avoid multiple collinearity in the models due to the mutual correlation among excess humidity, temperature and ventilation rate, the data were stratified according to the median indoor excess humidity. Here, water-based cleaning and repainting were considered PGE sources. Indoor excess humidity, temperature, ventilation rate in the child’s bedroom, and history of water damage were considered effect modifiers. We deemed that a significant interaction is present if the estimated effect of the given source on the PGE concentration differed by >30% between the strata. Subsequently, we formally tested the following respective interaction terms in the overall data to confirm the finding, (frequency of water-based cleaning × excess humidity), and (history of repainting × excess humidity).

## 3. Results

### 3.1. Pattern of Mean PGE Concentration, Compared to the Patterns of Terpenes, Texanols, BBzP and DEHP

Table 2 shows the pattern of geometric means of the four VOC groups according to the suspected sources and modifiers. The geometric mean of the PGEs linearly increased with water-based cleaning frequency, history of repainting at least one of the rooms at home, “newest” age of the surface material, “obviously poor” indoor air quality, impression of “obviously” stuffy and unventilated air by the building inspector, growing excess indoor humidity, and higher indoor temperature. Mean PGE levels in the families with hard surface floor (*i.e.*, linoleum, PVC, wood, or laminate) were about 4- fold higher (*P = 0.057*) than the families with the ‘other’ flooring material in the child’s bedroom.

The PGEs were weakly correlated with the total Texanol concentration *(Spearman’s coefficient = 0.205, p = 0.046),* and also with the total terpenes *(Spearman’s coefficient = 0.205, p < 0.001)*. On the other hand, in a sub-analysis of the homes with a reliable increase (≥25 mg) in dust weight, non-parametric correlations between PGEs with BBzP and DEHP were weaker (*Spearman’s coefficients = 0.144 and 0.070, p = 0.013 and 0.193*).

### 3.2. Comparisons of Trends in Compound Groups

Trend in the geometric mean concentrations of PGEs differed from those for the terpene hydrocarbons, BBzP, and DEHP, respectively (Table 2).

The mean terpenes and Texanols did not increase with higher frequency of water-based cleaning. The mean terpenes and Texanols were 7% and 25% higher in the homes with a history of repainting prior to or following the child’s birth, compared to those without similar history. Among those homes with hard floor surfaces (n = 383), the mean terpene concentrations were approximately two-times higher than the homes with other flooring type (n = 7). Age of the surface material in the child’s bedroom was not associated with any apparent trend in terpenes. In addition, mean Texanol levels was not associated with the age of the surface material, building type, history of water-damage, or any of the inspector’s assessment of the air quality.

Overall disparate patterns in geometric mean BBzP and DEHP concentrations were observed with behavioral and structural predictors (Table 2). While no apparent trend emerged for the BBzP levels according to the age of the surface material, “very old” material was associated with ~74% higher mean DEHP than the homes with “newest” material (*P-for-linear-trend = 0.027*). “Obviously poor” indoor air quality, but no other ratings, was linearly associated BBzP (*P-for-linear-trend = 0.028*) and DEHP (*P-for-linear-trend = 0.009*) compared to the homes with “good” air quality. Other characteristics, such as the type of the ventilation system, or the history of home flooding were not associated with notable differences in any of the compound groups.

### 3.3. Inspector Rating of Indoor Air Quality

The internal consistency of indoor air quality (IAQ) was high (Chronbach’s alpha = 0.89) among the three ratings (Table 2). In addition, excess indoor humidity was not significantly different for the homes with “obvious” rating than the “weak” rating in all three items. Also, the ventilation rate in the child’s room was almost identical for the “obvious” rated homes compared to the “weak” rated homes in all three items.

### 3.4. Modifiers of Indoor PGE Concentrations

**Figure 1:**
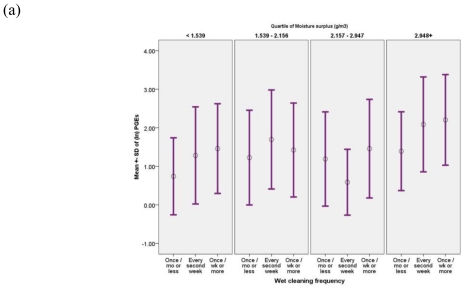

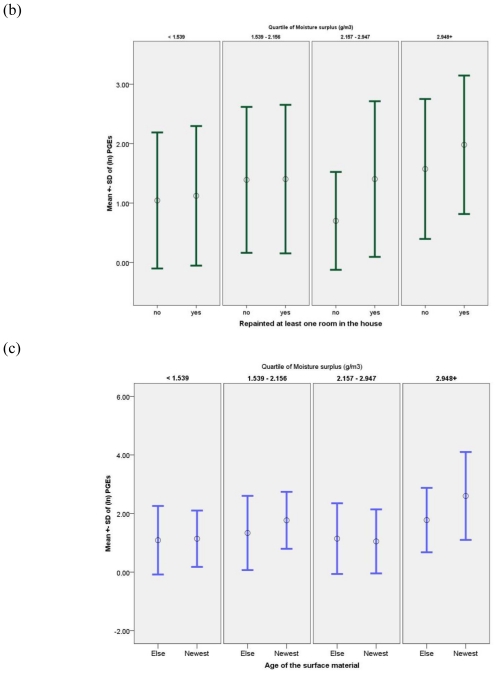
(a) Geometric mean PGEs (≤g/m^3^) associated with water-based cleaning frequency, according to excess indoor humidity (g/m^3^). (b) Geometric mean PGEs (≤g/m^3^) associated with repainting history, according to excess indoor humidity (g/m^3^). (c) Geometric mean PGEs (≤g/m^3^) associated with age of the surface material, according to excess indoor humidity (g/m^3^).

Excess indoor humidity (g/m^3^) was positively correlated with all groups of compounds. Figure 1 shows that the linear association between water-based cleaning and the mean PGEs in air is further enhanced by excess humidity. Geometric mean PGEs per each cleaning category was markedly higher within the highest humidity quartile (13.25, 12.23, and 5.86 ≤g/m^3^), compared to those within the lowest quartile (7.00, 5.82, and 3.60 ≤g/m^3^) (Figure 1(a)). Similarly, linear association in mean PGE concentration for those with repainting history was significantly greater within the highest quartiles of excess indoor humidity (10.85 *vs.* 6.68 ≤g/m^3^), compared to those within the lowest quartile (5.22 *vs.* 5.10 ≤g/m^3^) (Figure 1(b)). Furthermore, the mean PGEs for those with a “newest” surface material in the child’s room were augmented by excess indoor humidity (17.75 *vs.* 8.72 ≤g/m^3^ in the highest excess humidity category; 5.19 *vs.* 4.41 ≤g/m^3^ within the lowest category) (Figure 1(c)).

**Figure 2:**
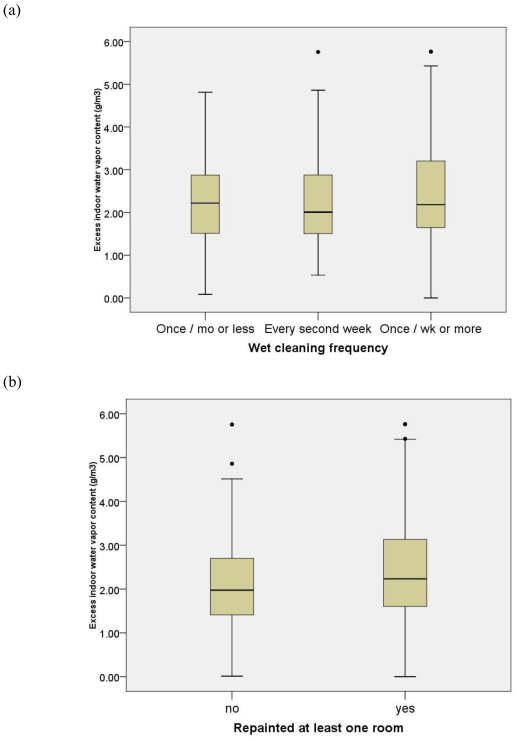

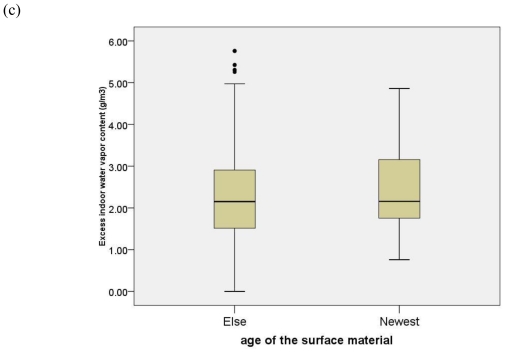
(a) Distribution of excess indoor humidity (g/m^3^) according to water-based cleaning frequency. (b) Distribution of excess indoor humidity (g/m^3^) according to repainting history. (c) Distribution of excess indoor humidity (g/m^3^) according to age of the surface material in the child’s bedroom.

At the same time, none of the suspected sources contribute to significantly elevated excess indoor humidity, demonstrating that humidity is unlikely to confound the source-PGE relationship (Figure 2).

### 3.5. Final Predictive Model

In order to clarify whether the PGEs, terpenes and the Texanols are emitted and/or modified by common factors, an identical predictive models were used to examine their ability to explain the variability in each group concentration (Table 3). The water-based cleaning > once/week was associated with a larger mean increase in PGEs (52%) for the homes with a high (≥2.158 g/m^3^) excess humidity, compared to the homes with similar frequency of wet-cleaning within low humidity homes (48%; *P-for-interaction* = 0.03). Furthermore, the history of repainting was associated >10-fold larger increase in PGE level in homes with a high excess humidity (63 % *vs.* 0.5% increase in mean PGEs, *P-for-interaction = 0.03*), compared to those with lower than median excess humidity. Quartile unit increase in the indoor temperature was associated with somewhat larger mean increase in the PGEs for those with a high excess humidity, compared to the low humidity homes (19 *vs.* 13%). Water -based cleaning every other week, but no other factors, was associated with a higher mean terpene level (41%) among the homes with high excess indoor humidity. No sources were associated with an increase in the Texanols (Table 3).

## 4. Discussion

The risks of VOC compounds emitted from cleaning agents, paints, and other surface material on the asthma and allergies remain controversial [1,13]. In our earlier analysis, PGEs in indoor air significantly predicted elevated risks of multiple allergic symptoms, and the diagnoses of asthma, rhinitis and eczema, respectively [15]. In addition, a unit PGE exposure was associated with an increased likelihood of IgE-sensitization. At the same time, a review of both epidemiologic and toxicological literature concluded that VOCs from cleaning and paints are likely to be mere correlates of biological allergens, combustion products, or dampness [13]. In part, the present analysis of VOCs and phthalates in the DBH study was conducted to further examine our earlier observation on PGE compounds and childhood asthma. Specifically, the sources and the correlations of the PGEs with other risk factors of asthma and allergies (*i.e.*, terpenes, Texanols, BBzP, and DEHP) were investigated here.

Building characteristics and occupant behaviors that contribute to elevated indoor PGE concentrations are markedly different from those of the terpenes, Texanols (Table 3) as well as BBzP and DEHP (Table 2). Such poor correlations suggest that the terpenes, BBzP, and DEHP are unlikely to confound the apparent associations of the PGEs with multiple allergic symptoms, and the diagnoses in our on-going DBH study.

In addition, significant augmentation of the PGEs in indoor air by excess humidity suggests that humidity might contribute to higher emission or retention of the PGEs [41]. The information regarding the history of repainting was collected 1.5 year prior to the present study. Also, all parents remained in the same house and have not changed most life-style practices during the 1.5 year period. To further validate this, the families (n = 18, 0.6% of cases and 1.1% controls) that renovated their house due to flooding were excluded before the onset of present investigation. A significant 63% (95% CI, 24–100%) increase in PGE concentration for those who repainted at least one of the rooms and also have a higher than median level of excess humidity suggests that PGE emission from paint might have been sustained far beyond several months period following the paint application. Such timeline supports the PGEs as risk factors, rather than mere correlates of parental allergen/adjuvant removal behavior following the clinical diagnosis of the child. Correlation structure among humidity, ventilation and temperature might additionally exert contemporary rather than long term relationship to PGE concentrations. An increase in excess humidity per unit reduction in ventilation rate was larger (2.40 g/m^3^) at the highest indoor temperature (21.7–25.6 °C), compared similar increase (0.97 g/m^3^/unit reduction in ventilation rate) at the lowest quartile (16.39–20.17 °C).

Lack of the association between the PGE sources examined here with excess indoor humidity demonstrates that humidity could not have confounded the sources—PGEs relationship (Figure 2). Rather, both water-based cleaning and repainting history significantly contribute to an increased PGE concentration in indoor air. Other experimental studies also demonstrated the water-based cleaning agents emit PGEs and terpenes [16].

PGEs, rather than limonene and terpenes are likely to be a significant risk factor of the asthma and allergy outcomes. This is supported by the persistence and the magnitude of the water-based cleaning’s association with the indoor PGE concentrations after accounting for other known predictors (Table 3). Other constituents of the cleaning agents, limonene and composite sum of the terpene hydrocarbons, were weakly correlated the PGEs (Spearman’s coefficient = 0.18 and 0.19, respectively). In the DBH study, limonene was neither an independent risk factor (adjusted-odds ratio (aOR), 1.15; 95% CI, 0.86–1.53) for the case status, nor a confounder of PGE-asthma/allergy associations. Similarly, the terpene hydrocarbons did not pose an independent risk on any of the outcomes, or confound the PGE-asthma/allergy associations. Another investigation, using only questionnaire of general domestic hygiene practice, observed somewhat elevated risks of higher home cleanliness with current wheezing symptoms (aOR, 1.16, 95% CI, 1.03–1.29) and current rhino-conjuntivitis (adjusted OR, 1.17, 95% CI, 1.04–1.31) in a large group of Australian children [42].

In the present DBH study, a large increase (41%) in PGE level for those families with “newest” surface material at home suggests that this might have additionally provided a long-term exposure. However, our present observation of the highest mean PGE concentration for the “newest” material requires a further investigation to clarify its constituent materials and to determine the exact emission patterns following the installation.

Consistent with our earlier analysis [43], PVC flooring material in the child’s bedroom (n = 188) was associated with 67% and 80% higher mean BBzP and DEHP levels, compared to those with linoleum flooring (n = 13). Multi-family houses were also more likely to use PVC flooring (n = 39) than the single-family house (n = 291) due to its strong correlation with socioeconomic position of the family [43]. The ventilation rate were also higher for the multifamily houses, compared to the single family house [43]. Linear increase in DEHP with higher ventilation rate suggests that DEHP transfer from vinyl flooring to dust occurs through the boundary layer [44]. Increased mixing of air might diminish the boundary layer and increase the DEHP transfer [44].

## 5. Conclusions

Use of water-based cleaning agent > once per month, repainting ≥ one room in the house, and “newest” surface material in the child’s bedroom was consistent with higher levels of PGEs in the child’s bedroom. Furthermore, the PGE levels in indoor air were significantly higher in homes with higher excess humidity in air and with higher temperatures. At the same time, these sources of PGE did not predict an elevated indoor humidity. Difference in specific sources and low correlations of the PGEs with other VOCs and the phthalates strengthen of the independence of PGE risks on the multiple allergic symptoms, and the respective diagnosis of asthma, rhinitis, and eczema.

## Figures and Tables

**Table 1:** Definition and distributions (μg/m^3^) of propylene glycol and glycol ethers (PGEs), terpene hydrocarbons, Texanols, and phthalates.

	N	Mean	S.D.	Min	25th	50th	75th	Max
	**Percentiles**							
**Propylene glycol and glycol ethers (CAS #)**								
1,2-propanediol (propylene glycol) (CAS # 57-55-6)	165	8.20	8.17	0.51	2.84	5.54	10.25	48.62
1-methoxy-2-propanol (α-isomer of Propylene Glycol Monomethyl Ether) (CAS # 107-98-2)	86	4.51	3.08	0.73	2.32	3.51	6.00	15.68
2-(2-butoxyethoxy)ethanol (CAS # 112-34-5)	69	4.73	4.90	0.46	1.95	2.87	6.04	30.33
1-butoxy-2-propanol (CAS # 5131-66-8)	65	7.15	8.87	0.60	2.21	4.03	8.08	53.04
2-(2-butoxyethoxy)ethanol acetate (CAS # 124-17-4)	33	3.30	3.05	0.53	1.62	2.32	3.57	13.26
2-butoxy ethanol (CAS # 111-76-2)	27	4.74	5.67	0.78	1.73	2.36	4.84	24.39
2-(2-(2-butoxyethoxy)ethoxy) ethanol (CAS # 143-22-6)	20	2.10	1.80	0.65	0.96	1.68	2.60	8.74
2-(2-ethoxyethoxy) ethanol (cas # 111-90-0)	16	9.72	9.39	2.26	4.38	5.73	11.59	36.86
1-(2-methoxypropoxy)-2-propanol (CAS # 13429-07-7)	11	6.63	5.74	1.39	2.73	3.99	13.91	16.15
Dipropylene glycol methyl ether (CAS # 34590-94-8)	7	4.43	2.52	1.83	2.55	3.74	6.35	9.16
2-(2-methoxyethoxy) ethanol (CAS # 111-77-3)	6	6.28	4.47	3.18	3.71	4.46	8.72	15.08
2-(2-hydroxypropoxy)-1-propanol (CAS # 106-62-7)	4	1.42	0.74	0.62	0.74	1.37	2.17	2.34
1-(2-methoxy-1-methylethoxy)-2-propanol (CAS # 20324-32-7)	3	6.66	2.48	4.13	4.13	6.76	9.08	9.08
1-propoxy-2-propanol (CAS # 1569-01-3)	2	5.23	5.20	1.55	1.55	5.23	8.91	8.91
2-(2-ethoxyethoxy) ethanol acetate (CAS # 112-15-2)	2	8.29	2.90	6.24	6.24	8.29	10.34	10.34
2,2-oxybis ethanol (Diethylene glycol) (CAS # 111-46-6)	1	7.97		7.97	7.97	7.97	7.97	7.97
Ethanediol (Ethylene glycol) (CAS # 107-21-1)	1	1.92		1.92	1.92	1.92	1.92	1.92
**Terpene hydrocarbons**								
γ–Terpinene (CAS # 99-85-4)	3	2.82	0.44	2.38	2.38	2.82	3.26	3.26
iso–Terpinolene (CAS # 586-62-9)	7	3.86	1.62	2.10	2.53	3.30	5.75	6.08
α–Terpinene (CAS # 99-86-5)	1	25.21	25.21	25.21	25.21	25.21	25.21	
α–Pinene (CAS # 80-56-8)	239	20.76	16.40	1.93	8.47	16.27	28.58	97.53
Limonene (CAS # 5989-27-5)	383	17.78	14.50	1.36	7.84	13.61	23.19	92.99
β–Pinene (CAS # 127-91-3)	35	4.41	2.58	0.62	2.77	3.85	5.18	13.21
Camphene (CAS # 79-92-5)	155	5.62	4.24	0.71	2.82	4.59	7.07	27.16
β–Phellandrene (CAS # 555-10-2)	14	5.81	4.34	1.39	2.37	4.97	8.08	16.35
**Texanol®**								
Texanol A	39	9.95	6.56	1.20	5.63	7.58	14.33	27.89
Texanol B	88	5.60	7.13	0.47	1.72	2.76	6.55	42.48
**Phthalate compounds in dust sample**a)								
n-butyl benzyl phthalate (BBzP)	296	0.38	2.64	0.01	0.09	0.16	0.30	45.55
di(2-ethylhexyl)phthalate (DEHP)	351	1.31	2.59	0.02	0.46	0.77	1.31	40.46

a) Sample size is reduced because only dust samples > 25 mg are considered [30].

**Table 2:** Geometric mean and geometric standard deviations of PGEs, terpenes, Texanols, BBzP and DEHP in the homes.

	PGEs		Terpenes		Texanols		BBzP a)b)		DEHP a)b)	
	nb)	GM ± GSD (μg/m^3^)	nb)	GM ± GSD (μg/m^3^)	nb)	GM ± GSD (μg/m^3^)	n	GM ± GSD (μg/m^3^)	n	GM ± GSD (μg/m^3^)
**Suspected Sources**										
Water-based cleaning frequency										
*P-for-linear-trend*		***0.046***		*0.800*		*0.522*		***0.048***		***0.026***
≥ Once / week	121	**5.40 ± 3.42**c)	121	21.14 ± 2.77	30	4.16 ± 3.79	95	0.18 ± 2.77	109	0.86 ± 2.68
Every other week	99	3.94 ± 3.51	99	24.25 ± 2.87	26	3.91 ± 2.46	72	0.17 ± 2.57	85	0.88 ± 2.72
≤ Once / month	165	3.13 ± 3.16	165	23.09 ± 2.34	38	4.71 ± 3.25	125	0.14 ± 2.42	151	0.68 ± 2.48
At least one room was repainted prior to/following the child’s birth										
*P-for-ANOVA*		***0.014***		*0.320*		*0.851*		*0.292*		*0.457*
Yes	247	4.28 ± 3.47	247	23.07 ± 2.62	66	4.64 ± 3.15	190	0.17 ± 2.67	224	0.80 ± 2.58
No	143	3.37 ± 3.18	143	21.49 ± 2.68	29	3.70 ± 3.18	106	0.15 ± 2.40	127	0.73 ± 2.68
Flooring material, child’s bedroom										
*P-for-ANOVA*		*0.110*		*0.382*		*0.178*		***0.000***		***0.002***
Linoleum	13	4.44 ± 3.89	13	22.77 ± 1.95	13	1.61 ± 1.03	12	0.12 ± 3.39	13	0.54 ± 2.98
PVC	211	4.42 ± 3.35	211	22.21 ± 2.64	211	4.25 ± 3.11	171	0.20 ± 2.46	188	0.95 ± 2.59
Wood	120	3.36 ± 3.43	120	24.44 ± 2.57	120	5.62 ± 3.40	82	0.11 ± 2.53	106	0.60 ± 2.42
Laminate	39	3.81 ± 3.11	39	21.25 ± 2.87	39	2.13 ± 1.71	25	0.16 ± 2.31	37	0.65 ± 2.81
Other ^d)^	7	1.32 ± 1.89	7	10.26 ± 3.41	7	6	0.13 ± 1.40	7	0.79 ± 1.75	
Age of surface materials in the child’s bedroom										
*P-for-linear-trend*		***0.049***		*0.556*		*0.073*		*0.727*		***0.027***
Very old	19	3.15 ± 3.23	19	21.82 ± 2.02	5	1.22 ± 2.16	15	0.17 ± 2.90	18	0.99 ± 1.98
Old	76	3.51 ± 3.31	76	26.04 ± 2.39	16	3.07 ± 3.22	56	0.16 ± 2.87	70	0.88 ± 2.92
Mixture of old/new	136	3.77 ± 3.35	136	20.69 ± 3.02	33	6.28 ± 2.77	104	0.15 ± 2.19	122	0.74 ± 2.47
Overall new	112	3.65 ± 3.31	112	21.96 ± 2.52	31	3.95 ± 3.02	90	0.17 ± 2.91	101	0.76 ± 2.72
Newest	38	6.42 ± 3.54	38	22.65 ± 2.54	7	5.60 ± 4.31	27	0.15 ± 2.11	33	0.57 ± 2.13
Building type										
*P-for-ANOVA*		*0.805*		*0.706*		*0.096*		*0.308*		*0.509*
Single family house	321	3.85 ± 3.31	321	23.16 ± 2.64	83	4.65 ± 3.17	243	0.16 ± 2.56	291	0.75 ± 2.57
Two family house	22	4.65 ± 4.27	22	21.52 ± 2.08	3	7.80 ± 4.20	16	0.15 ± 2.14	19	0.81 ± 2.43
Multifamily house	44	3.94 ± 3.50	44	18.69 ± 2.96	8	1.83 ± 2.01	34	0.15 ± 2.94	39	0.94 ± 3.09
Another	3	6.35 ± 4.73	3	18.76 ± 1.53	1	2.35 ± --	3	0.43 ± 1.38	2	0.49 ± 1.34
Secondhand smoke										
*P-for-ANOVA*		*0.755*		*0.394*		*0.355*		*0.421*		*0.632*
Yes	67	4.07 ± 3.16	67	20.34 ± 3.19	17	3.16 ± 3.09	55	0.17 ± 2.54	63	0.82 ± 2.58
No	313	3.87 ± 3.40	313	22.73 ± 2.52	75	4.59 ± 3.20	235	0.16 ± 2.60	280	0.77 ± 2.63
**Suspected Modifiers**										
Quartiles of excess indoor water vapor content over outdoor content (g/m^3^)										
*P-for-linear-trend*		***<0.001***		*0.137*		***0.031***		***0.007***		*0.165*
< 1.539	84	2.98 ± 3.18	84	21.28 ± 2.45	20	2.52 ± 2.77	65	0.12 ± 2.19	80	0.68 ± 2.61
1.539 - 2.156	84	4.04 ± 3.43	84	20.60 ± 2.75	18	5.09 ± 3.08	67	0.17 ± 2.17	82	0.78 ± 2.21
2.157 - 2.947	84	3.11 ± 3.30	84	18.92 ± 2.77	14	4.13 ± 4.06	59	0.16 ± 2.53	69	0.72 ± 2.91
≥ 2.948	84	**6.51 ± 3.24**c)	84	27.58 ± 2.39	30	5.79 ± 3.05	66	0.20 ± 3.45	75	0.88 ± 2.82
Quartiles of Temperature (°C)										
*P-for-linear-trend*		***0.003***		*0.279*		*0.323*		*0.064*		*0.589*
< 20.18	96	2.75 ± 2.90	96	19.95 ± 2.72	20	5.67 ± 2.53	71	0.13 ± 2.37	87	0.72 ± 2.72
20.18 - 20.96	98	4.86 ± 3.27	98	24.26 ± 2.45	24	4.21 ± 3.77	75	0.16 ± 2.36	87	0.84 ± 2.72
20.97 - 21.67	97	3.32 ± 3.36	97	21.10 ± 2.80	25	4.06 ± 3.46	71	0.17 ± 3.13	84	0.70 ± 2.69
≥ 21.68	98	**5.32 ± 3.64**c)	98	24.50 ± 2.56	25	3.92 ± 2.95	78	0.18 ± 2.47	92	0.83 ± 2.38
Quartiles of ventilation rate, child’s bedroom (air change / hr)										
*P-for-linear-trend*		*0.198*		*0.372*		*0.111*		*0.125*		***0.006***
< 0.220	94	4.64 ± 3.52	94	25.19 ± 2.49	24	6.28 ± 2.44	71	0.15 ± 2.25	82	0.69 ± 2.57
0.220 - 0.315	93	4.09 ± 3.48	93	22.16 ± 2.73	27	3.90 ± 3.05	76	0.14 ± 3.10	90	0.61 ± 2.78
0.316 - 0.435	94	3.65 ± 3.16	94	21.89 ± 2.42	22	4.23 ± 4.23	74	0.18 ± 2.53	86	0.85 ± 2.52
≥ 0.436	97	3.78 ± 3.40	97	22.19 ± 2.73	18	3.33 ± 3.24	69	0.17 ± 2.43	85	0.95 ± 2.44
Type of ventilation system at home										
*P-for-ANOVA*		*0.552*		*0.840*		***0.015***		*0.175*		*0.572*
Natural, and do not use a kitchen fan	20	2.81 ± 3.55	20	23.44 ± 1.83	8	3.43 ± 3.04	15	0.19 ± 3.02	18	0.75 ± 4.08
Natural and use a kitchen fan	229	4.29 ± 3.37	229	22.75 ± 2.85	62	5.09 ± 3.15	171	0.16 ± 2.57	211	0.76 ± 2.63
Exhaust air system	92	3.95 ± 3.62	92	20.80 ± 2.52	19	3.79 ± 3.03	73	0.17 ± 2.53	79	0.86 ± 2.49
Exhaust & supply air system	12	3.46 ± 3.02	12	15.91 ± 3.29	4	1.35 ± 1.61	9	0.16 ± 2.54	10	0.88 ± 1.93
Exhaust & supply with heat recovery	25	3.39 ± 2.82	25	27.41 ± 1.86	1	0.63 ± --	18	0.15 ± 2.63	21	0.74 ± 2.19
Other	7	1.76 ± 2.24	7	22.65 ± 1.73	7	0.08 ± 2.43	7	0.39 ± 2.52		
Water damage since spring 2000										
*P-for-ANOVA*		*0.915*		*0.813*		*0.110*		*0.764*		*0.625*
Yes	42	4.00 ± 3.77	42	21.94 ± 3.40	5	1.95 ± 2.65	33	0.15 ± 2.56	40	0.82 ± 3.15
No	344	3.91 ± 3.34	344	22.78 ± 2.55	89	4.56 ± 3.16	259	0.16 ± 2.60	306	0.76 ± 2.56
**Building Inspector Rating**										
First impression of stuffy or unventilated air										
*P-for-ANOVA*		***0.027***		***0.029***		*0.925*		*0.207*		*0.367*
Obvious	33	6.76 ± 3.81	33	31.77 ± 2.14	9	8.22 ± 3.29	26	0.20 ± 2.09	29	1.02 ± 2.00
Weak	85	3.79 ± 3.41	85	19.85 ± 3.08	18	8.65 ± 3.97	64	0.15 ± 2.79	75	0.85 ± 2.74
*P-for-ANOVA*		***0.027***		***0.029***		*0.925*		*0.207*		*0.367*
Obvious	33	6.76 ± 3.81	33	31.77 ± 2.14	9	8.22 ± 3.29	26	0.20 ± 2.09	29	1.02 ± 2.00
Weak	85	3.79 ± 3.41	85	19.85 ± 3.08	18	8.65 ± 3.97	64	0.15 ± 2.79	75	0.85 ± 2.74
Stuffy, earthy, or microbial smell										
*P-for-ANOVA*		*0.954*		*0.688*		*0.111*		*0.640*		*0.436*
Obvious	39	3.36 ± 3.21	39	25.38 ± 2.67	8	2.20 ± 2.56	30	0.15 ± 2.58	38	0.85 ± 2.85
Weak	47	3.31 ± 3.51	47	23.20 ± 2.92	11	5.80 ± 4.10	35	0.14 ± 2.48	42	0.70 ± 3.05
Chemical smell										
*P-for-ANOVA*		*0.451*		*0.950*		*0.137*		*0.865*		*0.429*
Obvious	13	2.47 ± 2.26	13	26.86 ± 3.53	1	3.84 ± --	7	0.14 ± 3.25	12	0.60 ± 4.13
Weak	29	3.41 ± 4.13	29	27.57 ± 3.44	3	22.33 ± 1.88	26	0.13 ± 2.57	27	0.84 ± 2.91

a) Sample size is reduced because only dust samples >25 mg are considered [30].

b) Reflects the number of respondents to given question.

c) The cut-off level of significant association for Bonferroni-corrected pair-wise comparison of specific categories was 0.00652. The cut-off value for the significant association for ANOVA was 0.05.

Includes three homes with wall-to-wall carpet and four homes with ‘other’ type.

**Table 3:** Predictive models of indoor PGEs, Terpenes, and Texanols.

		PGEs				Terpenes				Texanols			

		β	(95 % CI**)**		P	β	(95 % CI**)**		P	β	(95 % CI**)**		P
Excess humidity													
≤2.157 g/m^3^	y-intercept	−1.567	−4.507	1.373	0.294	3.977	1.707	6.247	0.001	5.667	0.083	−0.776	12.110
	**Wet-clean once/week**	**0.477**	**0.040**	**0.915**	**0.033**	−0.247	−0.585	0.091	0.151	−0.417	0.397	−1.408	0.574
	**Wet-clean every other week**	**0.581**	**0.131**	**1.031**	**0.012**	**0.406**	**0.059**	**0.753**	**0.022**	−0.420	0.443	−1.525	0.684
	Repainted ≥ one room	0.005	−0.367	0.376	0.980	0.074	−0.213	0.361	0.610	−0.357	0.429	−1.268	0.554
	“Newest” surface material	0.423	−0.195	1.042	0.178	0.148	−0.330	0.625	0.543	0.155	0.791	−1.029	1.338
	Temperature (quartile unit)	0.125	−0.017	0.267	0.084	−0.049	−0.159	0.061	0.380	−0.170	0.255	−0.470	0.129
	Ventilation rate in the child‘s bedroom (quartile unit)	−0.054	−0.222	0.113	0.523	0.004	−0.126	0.133	0.955	−0.149	0.432	−0.532	0.234
≥2.158 g/m^3^	y-intercept	−2.824	−6.290	0.642	0.110	0.398	−2.421	3.217	0.781	3.496	0.362	−4.189	11.181
	**Wet-clean once/week**	**0.519**	**0.100**	**0.938**	**0.015**	0.104	−0.236	0.444	0.547	0.246	0.594	−0.682	1.174
	Wet-clean every other week	0.217	−0.260	0.694	0.370	−0.138	−0.526	0.249	0.482	−0.285	0.547	−1.235	0.665
	**Repainted** ≥ **one room**	**0.630**	**0.243**	**1.017**	**0.002**	0.100	−0.215	0.415	0.532	0.016	0.971	−0.847	0.878
	“Newest” surface material	0.409	−0.195	1.013	0.183	−0.098	−0.589	0.393	0.695	2.386	0.089	−0.385	5.157
	**Temperature (quartile unit)**	**0.185**	**0.020**	**0.350**	**0.028**	0.127	−0.008	0.261	0.065	−0.064	0.725	−0.431	0.302
	Ventilation rate in the child‘s bedroom (quartile unit)	−0.081	−0.255	0.093	0.358	0.026	−0.116	0.167	0.718	−0.257	0.201	−0.658	0.143

## Appendix: Online Supporting Document

### Detection and Identification

An automated mass spectra library check (Wiley) gave the first preliminary identification results. Each of the library suggestions for component identification were then again cross checked against Norwegian Institute for Airway Research (NILU)’s database for indoor air pollutants which contains about 1,000 components. The database contains retention time indexes from compounds which were identified in indoor air samples at NILU on exact the same analytical system over the last 20 years. Most of the compounds within this database have been verified by direct injection of pure standard solutions or mixed standard solutions. The criteria for identification were over 80 % confidence match from the mass spectra library and a match to the retention time database within 5 seconds of relative retention together with a manual check of the retrieved mass spectrogram against the library mass spectrogram. If a peak did not meet those criteria it was named as “unidentified compound”.

### Calibration

The calibration was based on toluene equivalents [31,38,39]. Ten samples were run together with two standard injections before and after each series. Internal standards consisted of a solution containing 100 ng/≤L of benzene, toluene, ethylbenzene and xylenes in methanol [31,38,39]. Standard Tenax tubes were prepared by syringe onto the adsorbent followed by 5 minutes of dry nitrogen (20mL).

### Quality Assurance

Our VOC sampling approach with adsorption/thermal desorption coupled with gas chromatography-mass spectrometry (GC/MS) has been validated as sensitive, simple, and cost-effective assessment method [37,45,46]. Also, our sampling duration (60–90 min) was substantially longer than the standard protocol [28]. Compared to other methods, our present approach has an advantage of higher sensitivity [28]. In a number of indoor VOC investigations, which relied on Tenax TA as a general purpose adsorbent, overall very low inherent artifacts were observed [38]. Known artifacts of Tenax TA do not include glycol ethers. Thus, glycol ethers are unlikely to have been introduced in this investigation as sampling artifacts [28]. At the same time, no study as ever examined temporal stability of the 405 compounds in this study over time. A time period of 5 to 6 weeks between sampling and analysis could influence the artifact level and recovery rates. While this is likely to have biased the aromatic hydrocarbon level towards the null, there was no evidence that the PGEs were also influenced by the transport duration. The method used is in accordance to best laboratory practice and the recommendations given by DIN EN 14662-1 and Helmig 1996 [16].

The prevalence and concentration the VOCs detected in the present study are strikingly concordant with those detected from other Scandinavian countries. We compared the concordance of our detected VOCs with Finnish EXPOLIS study [47]. In the EXPOLIS study, air samples (2–3 L) from 183 homes were collected during the winter of 1996–1997 in Helsinki, Finland, focusing on 30 VOCs as target compounds. Extensive quality assurance and control standards were practiced. Of the 30 VOCs, 21 VOCs were also collected in our study. The prevalence (% detected in participant homes) of the VOC compounds were significantly correlated between the two studies (R^2^ = 0.57, *p < 0.001*) [15]. Also, eight VOC compounds, which were identified in ≥ 80% of the homes in both the studies (*i.e.*, toluene, limonene, hexanal, p/m-xylene, benzaldehyde, octanal, undecane, and ethylbenzene), their concentrations were significantly correlated (R^2^ = 0.612, *p = 0.022*). This suggests that compounds with low prevalence are also expected to have low concentrations in both DBH and EXPOLIS. For example, 2-methyl-1-propanol, observed in 5% of the homes of DBH study was 1.96 μg/m^3^ and 3.37 μg/m^3^ in EXPOLIS. Striking similarities in absolute concentration and correlation of the compounds between the two studies support the validity of our sampling and analytical procedures.

**Table 1:** Mean geometric concentration of individual PGE compounds according to excess indoor humidity and cleaning frequency.

	Excess Indoor Humidity (g/m^3^)					Cleaning frequency			
	N	GM	95%	CI		N	GM	95%	CI
1,2-propanediol									
<1.539	29	5.26	3.5	7.92	≥once/wk	64	6.12	4.85	7.73
1.539–2.156	35	4.77	3.56	6.39	Bi-wkly	34	5.94	4.45	7.92
2.157–2.947	30	5.96	3.93	9.03	≤once/mo	63	4.98	3.97	6.24
≥2.948	52	6.32	5.2	7.67		161			
	146								
1-methoxy-2-propanol									
<1.539	19	2.66	1.89	3.74	≥once/wk	29	4.27	3.45	5.3
1.539–2.156	18	4.21	2.8	6.33	Bi-wkly	26	3.85	2.91	5.09
2.157–2.947	15	3.54	2.58	4.85	≤once/mo	31	3.01	2.34	3.89
≥2.948	24	4.51	3.5	5.8		86			
	76								
2-(2-butoxyethoxy)ethanol									
<1.539	11	**1.51**	**0.8**	**2.83**	≥once/wk	34	3.2	2.44	4.2
1.539–2.156	10	**4.51**	**2.28**	**8.92**	Bi-wkly	14	4.42	2.67	7.33
2.157–2.947	13	**3.04**	**1.95**	**4.74**	≤once/mo	21	2.74	1.78	4.2
≥2.948	21	**4.30**	**3.16**	**5.83**		69			
	55								
1-butoxy-2-propanol									
<1.539	2	5.11	0	∞	≥once/wk	4	7.44	1.12	49.34
1.539–2.156	5	3.08	0.97	9.74	Bi-wkly	7	6.24	2.57	15.11
2.157–2.947	2	2.84	0	∞	≤once/mo	10	3.38	1.55	7.36
≥2.948	8	6.92	2.94	16.25		21			
	17								
2-(2-butoxyethoxy)ethanol acetate									
<1.539	7	1.43	0.8	2.58	≥once/wk	10	2.67	1.44	4.94
1.539–2.156	9	2.64	1.34	5.21	Bi-wkly	13	2.99	1.96	4.55
2.157–2.947	5	1.89	0.67	5.29	≤once/mo	10	1.67	0.98	2.85
≥2.948	9	3.63	2.08	6.33		33			
	30								
2-butoxy ethanol									
<1.539	2	1.7	0.03	109.77	≥once/wk	14	3.25	1.95	5.44
1.539–2.156	6	3.38	0.9	12.73	Bi-wkly	5	4.19	1.21	14.49
2.157–2.947	3	1.83	0.26	13.07	≤once/mo	8	2.29	1.21	4.36
≥2.948	13	3.67	2.31	5.82		27			
	24								
2-(2-(2-butoxyethoxy)ethoxy) ethanol									
<1.539	4	**1.62**	**1.12**	**2.34**	≥once/wk	10	1.98	1.15	3.4
1.539–2.156	3	**0.96**	**0.34**	**2.73**	Bi-wkly	2	2.2	0.05	102.27
2.157–2.947	3	**1.39**	**0.33**	**5.78**	≤once/mo	8	1.28	0.87	1.89
≥2.948	4	**3.64**	**1.21**	**10.98**		20			
	14								
2-(2-ethoxyethoxy) ethanol									
<1.539	3	8.07	2.43	26.79	≥once/wk	7	6.4	3.63	11.31
1.539–2.156	3	11.03	1.19	102.15	Bi-wkly	4	4.97	2.89	8.55
2.157–2.947	2	3.53	0.01	∞	≤once/mo	5	10.71	2.6	44.08
≥2.948	6	5.76	3.2	10.36		16			
	14								
1-(2-methoxypropoxy)-2-propanol									
<1.539	2	5.12	0.21	122.11	≥once/wk	4	4.43	0.99	19.81
1.539–2.156	3	4.94	0.31	79.13	Bi-wkly	3	7.76	1.58	38.18
2.157–2.947	1	3.52			≤once/mo	4	3.48	0.73	16.48
≥2.948	3	3.94	0.17	91.56		11			
	9								
Dipropylene glycol methyl ether									
<1.539	1	9.16			≥once/wk	2	3.67	2.95	4.58
1.539–2.156	2	4.02	0.01	∞	Bi-wkly	4	4.86	1.98	11.93
2.157–2.947	1	3.74			≤once/mo	1	1.83		
≥2.948	2	2.57	0.03	193.76		7			
	6								
2-(2-methoxyethoxy) ethanol									
<1.539	1	3.18			≥once/wk	1	15.08		
1.539–2.156	0				Bi-wkly	1	4.84		
2.157–2.947	1	3.88			≤once/mo	4	4.27	2.61	6.99
≥2.948	4	6.66	2.65	16.75		6			
	6								
2-(2-hydroxypropoxy)-1-propanol									
<1.539	1	0.62			≥once/wk	2	1.6	0.01	206.67
1.539–2.156	1	2.34			Bi-wkly	1	0.62		
2.157– 2.947	2	1.34	0.1	18.48	≤once/mo	1	1.65		
≥2.948	0					4			
	4								
1-(2-methoxy-1-methylethoxy)-2-propanol									
<1.539	1	9.08			≥once/wk	1	4.13		
1.539–2.156	1	6.76			Bi-wkly	2	7.84	1.21	50.92
2.157–2.947	1	4.13			≤once/mo	0			
≥2.948	0					3			
	3								
1-propoxy-2-propanol									
<1.539					≥once/wk	0			
1.539–2.156					Bi-wkly	1	1.55		
2.157–2.947					≤once/mo	1	8.91		
≥2.948						2			
2-(2-ethoxyethoxy) ethanol acetate									
<1.539	0				≥once/wk	0			
1.539–2.156	1	6.24			Bi-wkly	1	10.34		
2.157–2.947	0				≤once/mo	1	6.24		
≥2.948	1	10.34				2			
	2								
Texanol A									
<1.539	4	9.06	3.26	25.15	≥once/wk	12	9.56	5.9	15.49
1.539–2.156	9	7.27	4.49	11.77	Bi-wkly	9	6.07	3.16	11.66
2.157–2.947	6	7.12	3.49	14.52	≤once/mo	16	8.37	5.9	11.88
≥2.948	13	10.32	6.52	16.33		37			
	32								
Texanol B									
<1.539	19	1.99	1.43	2.77	≥once/wk	30	3.31	2.19	4.99
1.539–2.156	18	3.86	2.48	5.99	Bi-wkly	21	2.94	2.12	4.07
2.157–2.947	14	3.27	1.67	6.39	≤once/mo	35	3.7	2.64	5.19
≥2.948	28	**4.46**	**3.11**	**6.39**		86			
	79								

## References

[1] Jaakkola JJ Jaakkola MS Professional cleaning and asthma Curr. Opin. Allergy Clin. Immunol 2006 6 85 90 16520670 10.1097/01.all.0000216849.64828.55

[2] Bornehag CG Nanberg E Phthalate exposure and asthma in children Int. J. Androl 2010 33 333 345 20059582 10.1111/j.1365-2605.2009.01023.x

[3] Mendell MJ Indoor residential chemical emissions as risk factors for respiratory and allergic effects in children: A review Indoor Air 2007 17 259 277 17661923 10.1111/j.1600-0668.2007.00478.x

[4] Medina-Ramon M Zock JP Kogevinas M Sunyer J Torralba Y Borrell A Burgos F Anto JM Asthma, chronic bronchitis, and exposure to irritant agents in occupational domestic cleaning: a nested case-control study Occup. Environ. Med 2005 62 598 606 16109815 10.1136/oem.2004.017640 PMC1741089

[5] Medina-Ramon M Zock JP Kogevinas M Sunyer J Basagana X Schwartz J Burge PS Moore V Anto JM Short-term respiratory effects of cleaning exposures in female domestic cleaners Eur. Respir. J 2006 27 1196 1203 16510456 10.1183/09031936.06.00085405

[6] Wieslander G Norback D Edling C Airway symptoms among house painters in relation to exposure to volatile organic compounds (VOCS)—a longitudinal study Ann. Occup. Hyg 1997 41 155 166 9155237 10.1016/S0003-4878(96)00039-7

[7] Wieslander G Norback D Lindgren T Experimental exposure to propylene glycol mist in aviation emergency training: acute ocular and respiratory effects Occup. Environ. Med 2001 58 649 655 11555686 10.1136/oem.58.10.649 PMC1740047

[8] Wieslander G Norback D Nordstrom K Walinder R Venge P Nasal and ocular symptoms, tear film stability and biomarkers in nasal lavage, in relation to building-dampness and building design in hospitals Int. Arch. Occup. Environ. Health 1999 72 451 461 10541910 10.1007/s004200050398

[9] Kogevinas M Zock JP Jarvis D Kromhout H Lillienberg L Plana E Radon K Toren K Alliksoo A Benke G Blanc PD Dahlman-Hoglund A D’Errico A Hery M Kennedy S Kunzli N Leynaert B Mirabelli MC Muniozguren N Norback D Olivieri M Payo F Villani S Van Sprundel M Urrutia I Wieslander G Sunyer J Anto JM Exposure to substances in the workplace and new-onset asthma: An international prospective population-based study (ECRHS-II) Lancet 2007 370 336 341 17662882 10.1016/S0140-6736(07)61164-7

[10] Mazurek JM Filios M Willis R Rosenman KD Reilly MJ McGreevy K Schill DP Valiante D Pechter E Davis L Flattery J Harrison R Work-related asthma in the educational services industry: California, Massachusetts, Michigan, and New Jersey, 1993–2000 Am. J. Ind. Med 2008 51 47 59 18033692 10.1002/ajim.20539

[11] Obadia M Liss GM Lou W Purdham J Tarlo SM Relationships between asthma and work exposures among non-domestic cleaners in Ontario Am. J. Ind. Med 2009 52 716 723 19609981 10.1002/ajim.20730

[12] Zock J-P Plana E Jarvis D Anto JM Kromhout H Kennedy SM Kunzli N Villani S Olivieri M Toren K Radon K Sunyer J Dahlman-Hoglund A Norback D Kogevinas M The use of household cleaning sprays and adult asthma: An international longitudinal study Am. J. Respir. Crit. Care Med 2007 176 735 741 17585104 10.1164/rccm.200612-1793OC PMC2020829

[13] Nielsen GD Larsen ST Olsen O Lovik M Poulsen LK Glue C Wolkoff P Do indoor chemicals promote development of airway allergy? Indoor Air 2007 17 236 255 17542836 10.1111/j.1600-0668.2006.00468.x

[14] Emmen HH Muijser H Arts JHE Prinsen MK Human volunteer study with PGME: Eye irritation during vapour exposure Toxicol Lett 2003 140 141 249 259 10.1016/s0378-4274(03)00021-3 12676472

[15] Choi H Schmidbauer N Sundell J Hasselgren M Spengler J Bornehag CG Common household chemicals and the allergy risks in pre-school age children PLoS One 2010 5 e13423 20976153 10.1371/journal.pone.0013423 PMC2956675

[16] Singer BC Destaillats H Hodgson AT Nazaroff WW Cleaning products and air fresheners: Emissions and resulting concentrations of glycol ethers and terpenoids Indoor Air 2006 16 179 191 16683937 10.1111/j.1600-0668.2005.00414.x

[17] Wieslander G Norback D Ocular symptoms, tear film stability, nasal patency, and biomarkers in nasal lavage in indoor painters in relation to emissions from water-based paint Int. Arch. Occup. Environ. Health 2010 83 733 741 20549228 10.1007/s00420-010-0552-0

[18] Agency for Toxic Substances and Disease Registry (ATSDR) Toxicological Profile for 2-butoxyethanol and 2-butoxyethanol Acetate Department of Health and Human Services, Public Health Service Atlanta, GA, USA 1998 37676990

[19] Rumchev K Spickett J Bulsara M Phillips M Stick S Association of domestic exposure to volatile organic compounds with asthma in young children Thorax 2004 59 746 751 15333849 10.1136/thx.2003.013680 PMC1747137

[20] Wieslander G Norback D Venge P Changes of symptoms, tear film stability and eosinophilic cationic protein in nasal lavage fluid after re-exposure to a damp office building with a history of flooding Indoor Air 2007 17 19 27 17257149 10.1111/j.1600-0668.2006.00441.x

[21] Stewart RD Baretta ED Dodd HC Torkelson TR Experimental human exposure to vapor of propylene glycol monomethyl ether. Experimental human exposure Arch. Environ. Health 1970 20 218 223 5411392 10.1080/00039896.1970.10665577

[22] Wieslander G Norback D A field study on clinical signs and symptoms in cleaners at floor polish removal and application in a Swedish hospital Int. Arch. Occup. Environ. Health 2010 83 585 591 20407787 10.1007/s00420-010-0531-5

[23] Miller RR Hermann EA Young JT Calhoun LL Kastl PE Propylene glycol monomethyl ether acetate (PGMEA) metabolism, disposition, and short-term vapor inhalation toxicity studies Toxicol. Appl. Pharmacol 1984 75 521 530 6474479 10.1016/0041-008x(84)90188-1

[24] Ernstgård L Lof A Wieslander G Norback D Johanson G Acute effects of some volatile organic compounds emitted from water-based paints J. Occup. Environ. Med 2007 49 880 889 17693786 10.1097/JOM.0b013e3181161ced

[25] Wieslander G Norback D Bjornsson E Janson C Boman G Asthma and the indoor environment: the significance of emission of formaldehyde and volatile organic compounds from newly painted indoor surfaces Int. Arch. Occup. Environ. Health 1997 69 115 124 9001918 10.1007/s004200050125

[26] Jaakkola JJ Oie L Nafstad P Botten G Samuelsen SO Magnus P Interior surface materials in the home and the development of bronchial obstruction in young children in Oslo, Norway Am. J. Public Health 1999 89 188 192 9949747 10.2105/ajph.89.2.188 PMC1508530

[27] Jaakkola JJK Parise H Kislitsin V Lebedeva NI Spengler JD Asthma, wheezing, and allergies in Russian school children in relation to new surface materials in the Home Am. J. Public Health 2004 94 560 562 15054004 10.2105/ajph.94.4.560 PMC1448297

[28] Wolkoff P Schneider T Kildesø J Degerth R Jaroszewski M Schunk H Risk in cleaning: chemical and physical exposure Sci. Total Environ 1998 215 135 156 9599458 10.1016/s0048-9697(98)00110-7

[29] Järnström H Saarela K Kalliokoski P Pasanen AL Comparison of VOC and ammonia emissions from individual PVC materials, adhesives and from complete structures Environ. Int 2008 34 420 427 17997159 10.1016/j.envint.2007.09.011

[30] Bornehag CG Sundell J Weschler CJ Sigsgaard T Lundgren B Hasselgren M Hagerhed-Engman L The association between asthma and allergic symptoms in children and phthalates in house dust: a nested case-control study Environ. Health Perspect 2004 112 1393 1397 15471731 10.1289/ehp.7187 PMC1247566

[31] Klenø JG Wolkoff P Clausen PA Wilkins CK Petersen T Degradation of the adsorbent Tenax TA by nitrogen oxides, ozone, hydrogen peroxide, OH radical and limonene oxidation products Environ. Sci. Technol 2002 36 4121 4126 12380084 10.1021/es025680f

[32] Wolkoff P Clausen PA Wilkins CK Nielsen GD Formation of strong airway irritants in terpene/ozone mixtures Indoor Air 2000 10 82 91 11980106 10.1034/j.1600-0668.2000.010002082.x

[33] Engman L Bornehag CG Sundell J How valid are parents’ questionnaire responses regarding building characteristics, mouldy odour, and signs of moisture problems in Swedish homes? Scand. J. Public Health 2007 35 125 132 17454915 10.1080/14034940600975658

[34] Bornehag CG Sundell J Sigsgaard T Hagerhed-Engman L Association between ventilation rates in 390 Swedish homes and allergic symptoms in children: A nested case control study Indoor Air 2005 15 275 280 15982274 10.1111/j.1600-0668.2005.00372.x

[35] Nordtest, Ventilation: Local mean age of air—Homogenous emission techniques Nordtest method NT VVS 118 Nordtest, Finland 1997

[36] Bornehag CG Sundell J Lundgren B Weschler CJ Sigsgaard T Hagerhed-Engman L Phthalates in indoor dust and their association with building characteristics Environ. Health Perspect 2005 113 1399 1404 16203254 10.1289/ehp.7809 PMC1281287

[37] Wu CH Feng CT Lo YS Lin TY Lo JG Determination of volatile organic compounds in workplace air by multisorbent adsorption/thermal desorption-GC/MS Chemosphere 2004 56 71 80 15109881 10.1016/j.chemosphere.2004.02.003

[38] Helmig D Artifact free preparation, storage and analysis of solid adsorbent sampling cartridges used in the analysis of volatile Organic compounds in air J. Chromatogr. A 1996 732 414 417

[39] Uhde E Application of solid sorbents for the sampling of volatile organic compounds in indoor air Organic Indoor Air Pollutants: Occurrence-Measurement-Evaluation Wilhelm-Klauditz- Institut (WKl), Fraunhofer-Institut für Holzforschung Braunschweig, Germany 1999 Chapter 1

[40] Edwards RD Jurvelina J Saarela K Jantunena M VOC concentrations measured in personal samples and residential indoor, outdoor and workplace microenvironments in EXPOLISHelsinki, Finland Atmos. Environ 2001 35 4531 4543

[41] Chou MS Huang BJ Chang HY Degradation of gas-phase propylene glycol monomethyl ether acetate by ultraviolet/ozone process: A kinetic study J. Air Waste Manage. Assoc 2006 56 767 776 10.1080/10473289.2006.10464488 16805401

[42] Zhang G Spickett J Lee AH Rumchev K Stick S Household hygiene practices in relation to dampness at home and current wheezing and rhino-conjunctivitis among school age children Pediatr. Allergy Immunol 2005 16 587 592 16238584 10.1111/j.1399-3038.2005.00325.x

[43] Bornehag CG Sundell J Hägerhed-Engman L Sigsgaard T Association between ventilation rates in 390 Swedish homes and allergic symptoms in children Indoor Air 2005 15 275 280 15982274 10.1111/j.1600-0668.2005.00372.x

[44] Clausen PA Lindeberg Bille RL Nilsson T Hansen V Svensmark B Bowadt S Simultaneous extraction of di(2-ethylhexyl) phthalate and nonionic surfactants from house dust. Concentrations in floor dust from 15 Danish schools J. Chromatogr A 2003 986 179 190 12597625 10.1016/s0021-9673(02)02007-1

[45] Bayer CW Black MS Galloway LM Sampling and analysis techniques for trace volatile organic emissions from consumer products J. Chromatogr. Sci 1988 26 168 173 3379122 10.1093/chromsci/26.4.168

[46] Kuntasal OO Karman D Wang D Tuncel SG Tuncel G Determination of volatile organic compounds in different microenvironments by multibed adsorption and short-path thermal desorption followed by gas chromatographic-mass spectrometric analysis J. Chromatogr. A 2005 1099 43 54 16330271 10.1016/j.chroma.2005.08.093

[47] Edwards RD Jurvelin J Saarela K Jantunen M VOC concentrations measured in personal samples and residential indoor, outdoor and workplace microenvironments in EXPOLIS-Helsinki, Finland Atmos. Environ 2001 35 4531 4543

